# Coordinating the real‐time use of global influenza activity data for better public health planning

**DOI:** 10.1111/irv.12705

**Published:** 2019-12-03

**Authors:** Matthew Biggerstaff, Fredrick Scott Dahlgren, Julia Fitzner, Dylan George, Aspen Hammond, Ian Hall, David Haw, Natsuko Imai, Michael A. Johansson, Sarah Kramer, James M. McCaw, Robert Moss, Richard Pebody, Jonathan M. Read, Carrie Reed, Nicholas G. Reich, Steven Riley, Katelijn Vandemaele, Cecile Viboud, Joseph T. Wu

**Affiliations:** ^1^ Influenza Division Centers for Disease Control and Prevention Atlanta GA USA; ^2^ Global Influenza Programme World Health Organization Geneva Switzerland; ^3^ InQTel Inc Washington DC USA; ^4^ Department of Mathematics and School of Health Sciences University of Manchester Manchester UK; ^5^ MRC Centre for Global Infectious Disease Analysis School of Public Health Imperial College London London UK; ^6^ Division of Vector‐Borne Diseases Centers for Disease Control and Prevention San Juan PR USA; ^7^ Department of Environmental Health Sciences Mailman School of Public Health Columbia University New York NY USA; ^8^ Modelling and Simulation Unit Centre for Epidemiology and Biostatistics Melbourne School of Population and Global Health The University of Melbourne Melbourne Vic. Australia; ^9^ School of Mathematics and Statistics The University of Melbourne Melbourne Australia; ^10^ Immunisation and Countermeasures Division National Infection Service Public Health England London UK; ^11^ Centre for Health Informatics, Computing, and Statistics, Lancaster Medical School Faculty of Health and Medicine Lancaster University Lancashire UK; ^12^ Department of Biostatistics and Epidemiology University of Massachusetts Amherst MA USA; ^13^ Division of International Epidemiology and Population Studies Fogarty International Center National Institutes of Health Bethesda MA USA; ^14^ WHO Collaborating Center for Infectious Disease Epidemiology and Control School of Public Health The University of Hong Kong Hong Kong SAR China

**Keywords:** forecasting, incidence, influenza

## Abstract

Health planners from global to local levels must anticipate year‐to‐year and week‐to‐week variation in seasonal influenza activity when planning for and responding to epidemics to mitigate their impact. To help with this, countries routinely collect incidence of mild and severe respiratory illness and virologic data on circulating subtypes and use these data for situational awareness, burden of disease estimates and severity assessments. Advanced analytics and modelling are increasingly used to aid planning and response activities by describing key features of influenza activity for a given location and generating forecasts that can be translated to useful actions such as enhanced risk communications, and informing clinical supply chains. Here, we describe the formation of the Influenza Incidence Analytics Group (IIAG), a coordinated global effort to apply advanced analytics and modelling to public influenza data, both epidemiological and virologic, in real‐time and thus provide additional insights to countries who provide routine surveillance data to WHO. Our objectives are to systematically increase the value of data to health planners by applying advanced analytics and forecasting and for results to be immediately reproducible and deployable using an open repository of data and code. We expect the resources we develop and the associated community to provide an attractive option for the open analysis of key epidemiological data during seasonal epidemics and the early stages of an influenza pandemic.

Influenza infection causes substantial morbidity and mortality every year in populations worldwide,^1^ and occasional pandemics have the potential to cause far greater health impacts.^2^ The primary data sources used to track influenza are public health surveillance systems that report clinical visits for influenza‐like syndromes (including influenza‐like illness (ILI), acute respiratory infections (ARI) and severe acute respiratory infections (SARI)) during a given week and the numbers of positive virological tests for specific types and subtypes.^3^ Globally, the World Health Organization (WHO) curates two large public databases of syndromic case counts (FluID,^4^ since 2009) and virologically confirmed influenza infection (Flunet,^5^ since 1997), with timely and robust data for many countries (Figure 1).

**Figure 1:**
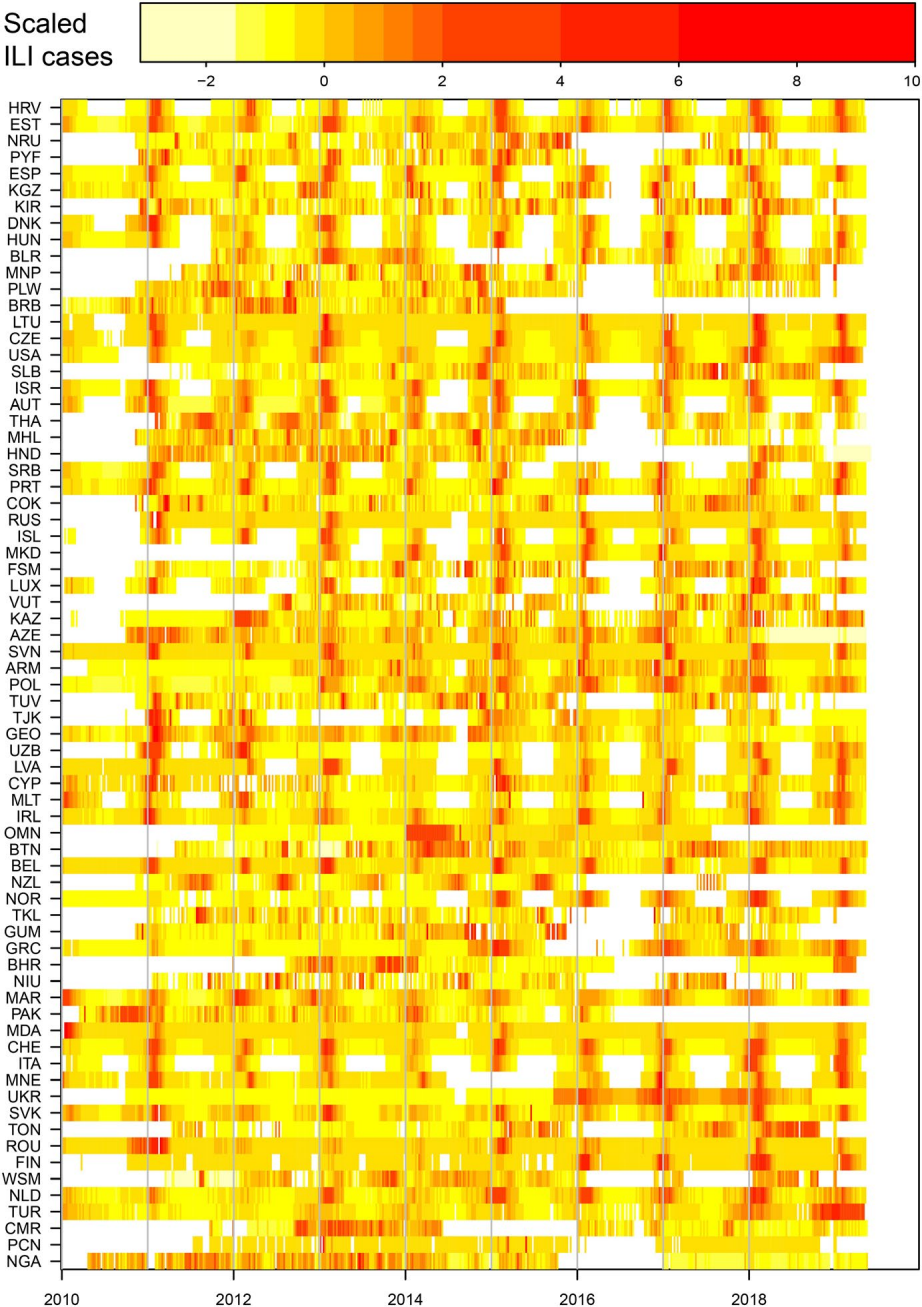
Influenza‐like‐illness (ILI) data from the FluID database. Weekly scaled rates of ILI since 2010 for all countries that have reported for at least 50% of weeks, from FluID database. For each country, ILI was rescaled by removing the mean and dividing by the variance. Colours represent the intensity of ILI activity from low (yellow) to high (red). Colours are based on the percentile of the distribution of scaled ILI (see above colour bar for values), with white representing lack of reporting. This plot can be reproduced by evaluating the R script “launch_figure.r” in the directory “notes” in the group code repository.^30^

Health planners must anticipate year‐to‐year and week‐to‐week variation in influenza activity when planning for and responding to pandemics or seasonal epidemics. For example, advanced analytics and modelling have been used to help inform pandemic planning^6^, ^7^, ^8^ and response.^9^, ^10^, ^11^ Also, retrospective analyses using data from surveillance systems and other studies have illuminated important features about the transmission dynamics of influenza, including the characterization of spatial incidence patterns,^12^, ^13^ planning for the optimal use of seasonal vaccines,^14^ early analyses of household transmission^15^ and global spatial spread.^16^

In addition, since the 2009 pandemic,^17^ quantitative models have been used to forecast seasonal influenza activity in real time. The field has grown rapidly^18^ from early retrospective studies^19^ to a large network of groups prospectively forecasting US influenza activity each year as part of a competition run by the US Centers for Disease Control and Prevention (CDC) to forecast the time of season onset, time of peak, intensity of peak and near‐term incidence.^20^ A number of different techniques are used in the competition including crowd‐sourced expert judgement forecasts,^21^, ^22^ mechanistic models,^19^ machine learning^23^ and statistical models.^24^ Also, different approaches can be combined into a single ensemble.^25^

However, despite this success in translating influenza surveillance data into actionable forecasts and a number of forecasting studies from other locations (eg Refs^26^, ^27^), there are currently no real‐time efforts to apply advanced analytics to influenza data at a global scale. Also, most public health agencies do not have resident expertise in advanced analytics such as infectious disease forecasting, nor the expertise to interpret those results. Accessing analytical capacity from within this group represents a resource for public health agencies. Generating actionable results from advanced analytics at local and global scales could provide unique insights for managing the consequences of seasonal and pandemic influenza.

Here, we describe the formation of the Influenza Incidence Analytics Group (IIAG), an informal WHO coordinated global effort to apply advanced analytics and modelling to these data in real‐time and thus provide additional insights to WHO Member States who report the data to WHO. The WHO IIAG network includes academics and public health officials from national, regional and global organizations. We organise regular open conference calls and maintain a repository with up‐to‐date global incidence data, accompanying housekeeping code, and initial analytical tools.

Our objective is to systematically couple data to analytical tools to improve the data and analytical capabilities where possible and, most importantly, to increase the quality of actionable real‐time influenza information available to public health institutions and organizations worldwide. With strong movements for both open data and open scientific code, we aim to gather both initiatives together to help create a community of investigators, developers and consumers of analysis.

The WHO IIAG teleconference calls have provided a forum for the exchange of ideas, identifying challenges and potential solutions between WHO staff, country public health practitioners and academic groups working on the advanced analytics and modelling of influenza. WHO staff and country representatives have presented current data and outlined specific questions of interest. In response, analytical and modelling groups have presented related works in progress and model‐derived forecasts based on the available WHO data. The frequent structured interactions between these communities have led to greater awareness and use of advanced analytics and modelling among public health experts, and greater understanding among the academic analytical community of public health challenges.

Analyses are presented and discussed. WHO staff report global patterns of incidence and issues emerging from different Members States, such as lower‐than‐ or higher‐than‐expected incidence for the time of year or unusual age patterns in reported cases. Individual countries can then present their own data and analyses; examples have included forecasting results from England and Wales, the United States (Figure 2A) and Australia (Figure 2B). We also have global analyses that have been undertaken using mechanistic models and publicly available WHO data (Figure 2C, Ref^28^).

**Figure 2:**
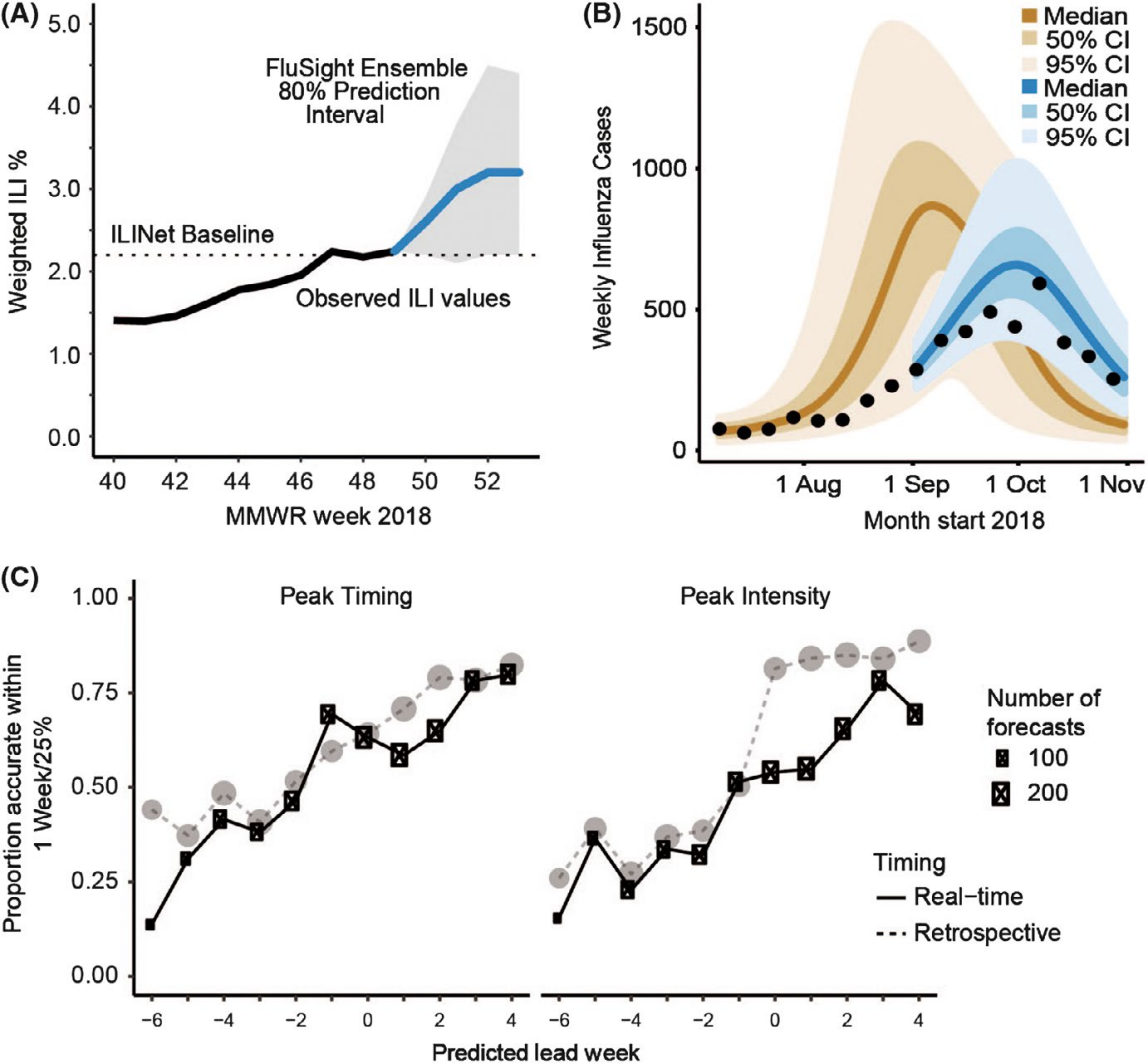
Examples of influenza forecasting results for the United States (A), Australia (B) and Europe (C). A, Forecast made for week 49, 2018 for national percentage of outpatient visits in the United States that would be for influenza‐like illness for the following 4 wks. Based on results from multiple groups.^25^ B, Forecasts of the number of laboratory‐confirmed influenza cases in Melbourne made on 2nd September 2018 (blue), with pre‐season “prior” forecast based also on data from previous seasons (brown, made 8th July 2018)^35^ (C) Real‐time forecast accuracy for 36 European countries during the 2017‐18 influenza season.^28^ Plots show the proportion of forecasts that accurately predicted peak timing within 1 week of the observed value (red) and that accurately predicted peak intensity within 25% of the observed value (blue). The *x*‐axis represents the number of weeks between the week of a forecast and the predicted peak week, with positive numbers indicating that the peak is predicted to have passed. The size of the points represents the number of forecasts produced at a given predicted lead week. Dashed lines show the comparative forecast accuracy when the forecasts are run retrospectively using the data available at the end of the season

The global data sets and accompanying example code are available as publicly available repositories^29^, ^30^ (Figure 1). The data are updated weekly, and the accompanying functions allow the rapid extraction of country‐level incidence time series for both ILI and virologically confirmed influenza.

While the initiative is new and seems productive, we have faced a number of challenges thus far. Firstly, data quality is not consistent among the reporting countries nor geographically representative. Timing, completeness and frequency of data reporting are not always optimal to allow forecasting. While the number of countries reporting data to FluNet (136 in 2016) and FluID (108 in 2016) has increased substantially over the years, there is still room for improvement.

In addition, any changes to internal reporting, participating sites, case definitions, etc, may not be apparent. Therefore, the interpretation of these data often requires some knowledge of the local context and thus input from local epidemiologists and public health professionals. Since these issues add an additional layer of complexity to real‐time forecasting, we have chosen not to launch a formal forecasting challenge at this time. We seek to augment data quality by working closely with countries to encourage frequent data updates and demonstrate the utility of collecting and reporting data which could then enable a formal forecasting exercise. Such an exercise, if successful, could provide further advocacy for data quality improvement efforts.

Current efforts are only possible because of the large community of analytical scientists who are independently supported to study influenza independent of IIAG. However, we are acutely aware we are not exploiting the full value of these data and that our current momentum likely cannot be sustained without dedicated support for forecasting groups and organizing capacity. Promisingly however, the WHO meeting on Using Influenza Data for Severity Assessment (November 2018) brought together country representatives, the Pandemic Influenza Special Studies working group and IIAG to discuss how to better integrate analytical tools developed for analysis of seasonal influenza with pandemic influenza assessment. The continued communication and trust building between these groups during seasonal epidemics will be critical in the realization of the benefits of advanced analytics and modelling to inform decision‐making in the next pandemic. This network would benefit greatly from modest central resources as previously demonstrated for prior networks of analytical scientists.^31^, ^32^

The early stages of an influenza pandemic are a special case of an infectious disease outbreak:^33^, ^34^ an outbreak which is more likely to occur than other pathogen types and which is also relatively likely to result in a large epidemic. Many national influenza pandemic plans (eg United States, UK, Australia, Japan) articulate enhanced data collection protocols, the use of routine surveillance systems for non‐routine purposes, and similar, to assess pathogen characteristics such as transmissibility and severity in the early stages of the pandemic. We would expect our activities and resources to continue in the event of an influenza pandemic. We would hope to provide an attractive option for the open analysis of key epidemiological data during the first few weeks. Developing these collaborative working groups during “peacetime” (ie not in the early stages of a real pandemic) is critical to improving the ways in which advanced modelling is integrated into public health decision‐making. We articulate a strategic vision and provide a prototype technical implementation for bringing together global expertise on infectious disease surveillance and real‐time modelling. These efforts provide an important template for informing and supporting public health efforts to combat global outbreaks of infectious disease.

## DISCLAIMER

The findings and conclusions in this report are those of the authors and do not necessarily represent the official position of the Centers for Disease Control and Prevention.
